# Assessment of knowledge regarding tuberculosis among non-medical university students in Bangladesh: a cross-sectional study

**DOI:** 10.1186/s12889-015-2071-0

**Published:** 2015-07-28

**Authors:** Masud Rana, Abu Sayem, Reazul Karim, Nurul Islam, Rafiqul Islam, Tunku Kamarul Zaman, Golam Hossain

**Affiliations:** Department of Population Science & Human Resource Development, University of Rajshahi, Rajshahi, 6205 Bangladesh; Divisional TB Expert, National Tuberculosis Control Program, Directorate General of Health Services, Dhaka, Bangladesh; Development Association for self-reliance, Communication and Health, Rajshahi, 6201 Bangladesh; Department of Statistics, University of Rajshahi, Rajshahi, 6205 Bangladesh; National Orthopaedic Centre of Excellence for Research and Learning (NOCERAL), Department of Orthopaedic Surgery, University of Malaya, Kuala Lumpur, Malaysia

**Keywords:** Tuberculosis, Knowledge, Non-medical university students, Socio-economic factors, *χ*^2^-test

## Abstract

**Background:**

Tuberculosis (TB) is the second leading cause of human death and TB is one of the major public health problems in Bangladesh. The aim of the present study was to assess the Knowledge about TB among non-medical university students in Bangladesh.

**Methods:**

A cross-sectional survey was performed on 839 non-medical university students. Data were collected from University of Rajshahi from March to August 2013 using a standard semi-structured questionnaire. Chi-square test was utilized to find the factors which are associated with students’ knowledge about TB.

**Results:**

Among 839 students, male and female were 68.2 % and 31.8 % respectively. Most of the students (94.4 %) were informed about the term TB, among them 50 % got information from electronic media. More than 50 % students believed that TB is a communicable disease, 42.8 % students agreed that bacteria is an agent for TB, most of the subjects (93 %) had the knowledge about the vaccination against TB and 97.6 % students believed that TB is curable. However, students had poor knowledge about latent TB (13.7 %) and DOTs program (28.5 %). *χ*^2^-test demonstrated that gender, residence, type of family and parents education were associated with students’ knowledge of TB.

**Conclusion:**

In the present study demonstrated that the level of general knowledge about TB was insufficient among non-medical university students. Consequently, health education program is needed to improve the knowledge among university students regarding TB.

## Background

In 1993, World Health Organization (WHO) declared that tuberculosis (TB) is a global emergency as because more than two billion people in the world is infected with mycobacterium tuberculosis, and they have the chance of developing TB at any stages of life. Furthermore, the infection rate is higher in developing countries like Bangladesh. TB is the second leading cause of death after human immune deficiency virus (HIV), as the greatest killer worldwide due to a single infectious agent [1]. Every year, more than 1.3 million people die around the world due to TB [2]. 82 % deaths occur in 22 high burden countries (HBCs) and Bangladesh rank 6^th^ among HBCs. Low awareness, education, income and high population density, smoking, diabetes, certain drugs and other associated diseases are the contributory factors for TB in Bangladesh [1]. The Government of Bangladesh, under the Ministry of Health and Family Welfare, Directorate General of Health Services (DGHS) are providing free diagnostic and treatment support to all irrespective of age, race, sex, occupation, geography or education through its National Tuberculosis Control Program (NTP). The Government of Bangladesh is also conducting different sessions, seminars, workshops, meeting, conferences, orientation on TB from 1993 using mass media and other media to aware the people at all level. Though Bangladesh has adopted internationally recommended Directly Observed Treatments Short Course (DOTs) strategy in 1993 [1] and STOP TB strategy in 2006 for effective control of TB in Bangladesh and also achieved more than 92 % treatment success rate through public private partnership and involvement of huge health care providers [1]. Still it requires more attention on knowledge and awareness of the community people regarding many areas; especially about diagnostic and treatment service opportunities, availability of services, mode of transmission, effect as well as impact of disease, DOTs, type of disease etc. Without proper diagnosis in early stage and irregularity of treatment can be a serious threat for the country as a whole due to chance of increasing spread and development of drug resistant (DR) TB. The DR-TB is a double burden for the society due to more duration of sufferings, high treatment cost and more chance of deaths. People at all levels need to be aware for early health seeking and regularity of treatment. Several studies have surveyed the knowledge about TB and they reported that poor knowledge, attitudes and practices regarding TB among general population [3, 4], among pre-university students [5, 6], high and secondary school students [7, 8]. Most of the surveys took place among medical students, health care workers and other occupation [9–13]. However, some researchers have surveyed the knowledge about TB among non-medical university students in other countries [14, 15]. We assume that a significant portion of middle and higher educated people have poor knowledge on TB including non-medical university students in Bangladesh. On the other hand, there is no survey on level of knowledge and awareness about TB among non-medical university students in Bangladesh. Particularly with respect to Bangladeshi populations one study has surveyed the knowledge about TB transmission among ever-married women in Bangladesh [16]. Another study has surveyed knowledge about TB in two areas in northern Bangladesh to assess and compare the level of knowledge, attitude and practice towards leprosy and TB among two communities [17]. Special attention should be paid to university students considering their potential influence on the family and their contribution to the nation’s workforce in near future in a particular nation. Due to their unique role in near future in the society, it is important to investigate the knowledge about communicable disease like TB. Considering the level of knowledge on common symptoms, disease agent, type of disease, latent infection, source of gaining knowledge, vaccination against TB, treatment system is fundamental information about TB which is necessary to analyze. Association between the knowledge about TB among non-medical university students and socio-economic factors such as gender, religion, residence, parents’ education, occupation and income, type of family, in order to ensure corrective measures can be undertaken.

Therefore, the objective of this study is to assess the knowledge about TB among non-medical university students in Bangladesh.

## Methods

The cross-sectional study sample consisted of 839 (male 572 and female 267) university students residing in student halls at the University of Rajshahi, Bangladesh. The University of Rajshahi is a non-medical and the second largest university in Bangladesh, with students coming from all over the country. The university has sixteen (11 for male and 5 for female) halls of residence accommodating a total of 25000 students (20000 male and 5000 female) at any particular time. Students were interviewed from March to August 2013 using a semi-structured questionnaire containing pre-coded and open-ended questions. The questionnaire had five different parts: (i) general information, (ii) demographic characteristics, (iii) parents’ socio-economic characteristics, (iv) general and specific knowledge about TB. This study was approved by National Tuberculosis Control Program (NTP), DGHS Bangladesh and BMRC. We received written consent from all subjects in this study.

### Sample size determination

Since our target population is known (25000 students), the following formula has been used for calculating sample size:

$n=\frac{N}{1+N{d}^2}$, where *n* = required sample size, *N* = population size (in here 25000), d = marginal error (we considered, d = 0.033), 95 % confidence level has been considered. The formula provided that the significant sample size was 839 for this study.

### Sampling technique and analysis

Since our population was heterogeneous and population divided into different groups (male and female), within the group are homogeneous, stratified random sampling with a proportional allocation technique was used for selecting sample from University of Rajshahi, Bangladesh.

The two items ‘editing and coding’ were processed in computer. Microsoft Excel and Microsoft Word were also used. *χ*^2^-test was utilized in this study to find the association between knowledge about TB and some selected socio-economic and demographic factors. The data was analyzed with Statistical Package for Social Sciences (SPSS) version 17.0. A value of p < 0.05 was regarded as statistically significant in the analysis.

## Results

In the present study, we surveyed the knowledge about TB of 839 university students, among them 572 (68.2 %) and 267 (31.8 %) were male and female, respectively. 70.4 % and 29.6 % students came from rural and urban area respectively. More than 94 % students declared that they knew the term TB and 5.6 % were confused about the term. Among the subjects 43.7 %, 18.4 %, 23.7 % and 14.2 % were first time informed about the term TB from electronic media, print media, relatives and friends and other sources respectively.

More than 30 % students perceived that TB is as non-communicable disease and 14.8 % students answered that they did not have idea about the term communicable and non-communicable diseases regarding TB. However, 51.5 % respondents answered properly that TB is a communicable disease. The knowledge of communicable disease regarding TB, male had higher percentage (53.7 %) than female (46.8 %) and *χ*^2^-test demonstrated that the association between the knowledge of type of TB disease and gender was statistically significant (p < 0.05). The respondents who came from rural environment had higher knowledge (53.1 %) than urban (47.6 %) and we noticed that the association between the type of TB disease and residence was significant (p < 0.05). Students who came from single family had more knowledge (52.2 %) about the communicable disease of TB than their counterparts (48.8 %) and the association between the type of TB disease and type of family was significant (p < 0.05) (Table 1).

**Table 1:** Knowledge about type of TB disease and its association with socio-economic and demographic factors

		Is TB communicable disease?				
Variables	Group	Yes, 432 (51.5 %)	No, 283 (33.7 %)	No idea, 124 (14.8 %)	*χ* ^2^-value	p-value
Religion	Muslim	383 (50.9 %)	255 (33.9 %)	114 (15.2 %)	1.218	0.544
	Hindu	49 (56.32 %)	28(33.7 %)	10(12.0 %)		
Gender	Male	307 (53.7 %)	178 (31.1 %)	87 (15.2 %)	5.52	0.043
	Female	125 (46.8 %)	105 (39.3 %)	37 (13.9 %)		
Parents’ education	No Education	71(53.8 %)	38(28.8 %)	23(17.4 %)	7.25	0.298
	Primary	69(50.4 %)	45(32.8 %)	23(16.8 %)		
	Secondary	105(52.5 %)	61(30.5 %)	34(17.0 %)		
	Higher	187(50.5 %)	139(37.6 %)	44(11.9 %)		
Residence	Rural	314(53.1 %)	182(30.8 %)	95(16.1 %)	8.42	0.015
	Urban	118(47.6 %)	101(40.7 %)	29(11.7 %)		
Type of family	Single Family	349(52.2 %)	231(34.5 %)	89(13.3 %)	5.79	0.045
	Joint Family	83(48.8 %)	52(30.6 %)	35(20.6 %)		

Among the respondents, 42.8 % believed that TB is a bacterial disease, while others respondents had wrong or no perception about the disease agent. Among the students, female had higher knowledge (48.7 %) about the agent of TB than male (40.0 %) and we observed that the association between knowledge about the agent of TB and gender was significant (p < 0.05). Respondents who came from single family had more knowledge (44.8 %) about the disease agent of TB than their counterparts (34.7 %) and the association between these two factors was significant (p < 0.05) (Table 2).

**Table 2:** Knowledge about agent of TB and its association with socio-economic and demographic factors

		Which agent is responsible for TB disease?					
Variables	Group	Bacteria, 359 (42.8 %)	Virus, 272 (32.4 %)	Other, 18 (2.1 %)	Don’t know, 190 (22.6 %)	*χ* ^2^-value	p-value
Religion	Muslim	320(42.6 %)	239(41.8 %)	17(2.3 %)	176(23.4 %)	3.294	0.349
	Hindu	39(44.9 %)	33(37.9 %)	01(1.2 %)	14(16.1 %)		
Gender	Male	229(40.0 %)	185(32.3 %)	14(2.4 %)	144(25.2 %)	9.030	0.029
	Female	130(48.7 %)	87(32.6 %)	4(1.5 %)	46(17.2 %)		
Parents’ education	No Education	48(36.4 %)	47(35.6 %)	2(1.5 %)	35(26.5 %)	11.992	0.214
	Primary	53(38.7 %)	42(30.7 %)	4(2.9 %)	38(27.7 %)		
	Secondary	79(39.5 %)	68(34.0 %)	5(2.5 %)	48(24.0 %)		
	Higher	179(48.4 %)	115(31.1 %)	7(1.9 %)	69(18.6 %)		
Residence	Rural	238(40.3 %))	201(34.0 %)	15(2.5 %)	137(23.2 %)	6.213	0.102
	Urban	121(48.8 %)	71(28.6 %)	3(1.2 %)	53(21.4 %)		
Type of family	Single Family	300(44.8 %)	213(31.8 %)	13(1.9 %)	143(21.4 %)	6.583	0.046
	Joint Family	59(34.7 %)	59(34.7 %)	5(2.9 %)	47(27.6 %)		

More than 75 % respondents believed that TB is a major public health problem as it spread from person to person through coughing or sneezing. Female students (83.5 %) had higher perception about TB as a public health problem than male (75.0 %), and the association between the knowledge about TB as public health problems and gender was highly significant (p < 0.01). Students whose father’s were service holder had more knowledge (81.4 %) than their counterparts and the association between the knowledge about TB as public health problem and father occupations was significant (p < 0.01).

Regarding BCG vaccine against TB in children, most of the respondents (93 %) were aware about the vaccination of TB, among them, female (76.8 %) were more aware than male (63.8 %). We noticed that the association between the knowledge about the vaccination of TB and gender was statistically significant (p < 0.01).

More than 97 % respondents strongly believed that TB is a curable disease, among them, male and female were 96.7 % and 99.6 % respectively. The association between the knowledge about curable and gender was significant (p < 0.01). Higher educated parents’ son/daughter (99.2 %) had more knowledge than secondary (96.5 %), primary (98.5 %) and uneducated (93.9 %), and the association between knowledge about TB as a curable disease and parents’ education was significant (p < 0.01) (Table 3).

**Table 3:** Knowledge about the TB as curable disease and its association with socio-economic and demographic factors

		Do you think about TB as curable disease?			
Variables	Group	Yes 819 (97.6 %)	No 20 (2.4 %)	*χ* ^2^-value	p-value
Religion	Muslim	733(97.5 %)	19(2.5 %)	0.636	0.425
	Hindu	86(98.9 %)	01(1.1 %)		
Gender	Male	553(96.7 %)	19(3.3 %)	6.795	0.009
	Female	266(99.6 %)	01(.4 %)		
Parents’ education	No Education	124(93.9 %)	08(6.1 %)	13.176	0.004
	Primary	135(98.5 %)	02(1.5 %)		
	Secondary	193(96.5 %)	07(3.5 %)		
	Higher	367(99.2 %)	03(.8 %)		
Residence	Rural	578(97.8 %)	13(2.2 %)	0.291	0.589
	Urban	241(97.2 %)	07(2.8 %)		
Type of family	Single Family	653(97.6 %)	16(2.4 %)	0.001	0.976
	Joint Family	166(97.6 %)	4(2.4)		

Students were asked for latent TB as more complex question and only 13.7 % answered positively while 86.3 % respondents did not have idea about latent TB. Male students had higher knowledge (15.4 %) than female (10.1 %), and the association between the knowledge about latent TB and gender was significant (p < 0.05). Students who were smoker had more knowledge (22.5 %) than their counterparts (12.8 %), and the association between the knowledge about latent TB and smoking status was significant (p < 0.05) (Table 4).

**Table 4:** Knowledge about latent TB and its association with socio-economic and demographic factors

		Do you know about latent TB?			
Variables	Group	Yes, 115 (13.7 %)	No, 724 (86.3 %)	*χ* ^2^-value	p-value
Religion	Muslim	103(13.7 %)	649(86.3 %)	0.001	0.980
	Hindu	12(13.79 %)	75(86.21 %)		
Gender	Male	88(15.4 %)	484(84.6 %)	4.28	0.039
	Female	27(10.1 %)	240(89.9 %)		
Parents’ education	No Education	15(11.4 %)	117(88.6 %)	2.41	0.492
	Primary	16(11.7 %)	121(88.3 %)		
	Secondary	33(16.5 %)	167(83.5 %)		
	Higher	51(13.8 %)	319(86.2 %)		
Residence	Rural	83(14.0 %)	508(86.0 %)	.192	0.661
	Urban	32(12.9 %)	216(87.1 %)		
Type of family	Single Family	94(14.1 %)	575(85.9 %)	0.330	0.565
	Joint Family	21(12.4 %)	149(87.6 %)		

The knowledge on treatment system through DOTs was surveyed among students. More than 90 % of students knew about DOTs while 8.6 % did not have knowledge about treatment system through DOTs. Hindu students had higher knowledge (37.9 %) about DOTs than Muslim (27.4 %), and we observed that the association between knowledge about DOTs and religion was significant (p < 0.05). Male students had more knowledge (30.9 %) than female (23.2 %), the association between the knowledge about DOTs and gender was significant (p < 0.05). Students who came from single family had more knowledge (30.2 %) than their counterparts (21.8 %). We noticed that the association between the knowledge about DOTs and type of family was significant (p < 0.05) (Table 5).

**Table 5:** Knowledge about DOTs program and its association with socio-economic and demographic factors

		Do you know about DOTs Program?			
Variables	Group	Yes, 767 (91.4 %)	No, 72 (8.6 %)	*χ* ^2^-value	P-value
Religion	Muslim	206(27.4 %)	546(72.6 %)	4.250	0.039
	Hindu	33(37.9 %)	54(62.1 %)		
Gender	Male	177(30.9 %)	395(69.1 %)	5.330	0.021
	Female	62(23.2 %)	205(76.8 %)		
Parents’ education	No Education	40(30.3 %)	92(69.7 %)	0.354	0.950
	Primary	38(27.7 %)	99(72.3 %)		
	Secondary	58(29.0 %)	142(71.0 %)		
	Higher	103(27.8 %)	267(72.2 %)		
Residence	Rural	177(29.9 %)	414(70.1 %)	2.101	0.147
	Urban	62(25.0 %)	186(75.0 %)		
Type of family	Single Family	202(30.2 %)	467(69.8 %)	4.728	0.030
	Joint Family	37(21.8 %)	133(78.2 %)		

## Discussion

In the present study, we surveyed the knowledge among non-medical university students about TB, particularly on common symptoms [1], disease agent, communicable or non-communicable, latent TB, vaccination against TB, treatment system etc. This study also examined the perception of respondents about TB as a public health problem, source of gaining knowledge, urban–rural and male–female ratio, parents’ education and income etc. Most of the students declared that they heard the term TB from various sources, such as electronic media, print media, relatives and friends. Similar results were found among other people in Rajshahi city including students [18]. In the current study demonstrated that nearly half of the students gathered knowledge about TB from electronic media, and it was a strong media to communicate people at all level to aware, inform and guide them for receiving information and services with free of cost from their nearest distance. Friends and relatives were also playing a vital role in disseminating information to others. In addition, parent’s education have played a significant role to increase their children’s knowledge about communicable disease such as TB. Different sessions, meetings, workshops, drama, rally was influencing others as discussion matter. Printed media, bill boards, leaflets; posters were also important media to inform about TB. In the present study have shown that nearly half of the students properly knew that TB is a communicable disease and it spread from person to person. A remarkable number of students had no knowledge on TB as communicable disease. It indicated that less involvement of students in TB related sessions, meetings or workshops. Though government as well as its several partner NGOs were working for advocacy, communication and social mobilization (ACSM) in Rajshahi district, they needed to perform adequate sessions or meeting with non medical university students. We found that the students who came from rural area had more knowledge regarding communicable disease than their counterpart. This might be happened due to frequent advertisement about communicable disease through Bangladesh television (BTV) which was largely observed by rural people. The urban people usually enjoyed other television channels like dish television. Now a day, dish TV channels are expanding to rural areas of Bangladesh. So, it is necessary to widely utilize dish TV channels as electronic media for TB related advertisement. Poverty, illiteracy, higher fertility has higher chance of developing TB. Once TB develops in any family, all members of the family become experienced and gained knowledge about symptoms, services and management of TB. In this study, nearly half of the students believed that TB is a bacterial disease while other had no knowledge or wrong perception. It may indicate less inclusion of TB related disease idea in general education curriculum. In developing countries like Bangladesh, more than half of whole populations are infected with TB as a latent TB. This latent TB has the chance of reactivation to active disease at any time when environment is favorable. But unfortunately the knowledge about latent TB among students was very poor. Poor nutrition, diabetes or immunosuppressive drugs, smoking or certain diseases can reactivate the latent TB. University students should know more about latent TB and its environmental condition. In the present study demonstrated that male had more aware than female about the knowledge on TB disease, latent TB and DOTs, on the other hand, female had more knowledge on disease agent and its curable than male. This gender difference may indicate the cultural and life styles in Bangladesh. The most of the time, mother as well as daughter usually stay at home and more prone to enjoy TV from where they receive limited information. Comparatively they are less exposed to market or public places. On the other hand, males are more exposed to markets, hospitals and other important public places. So, they have more chance to gain information from various sources such as bill boards, posters and friends along with TV.

In case of type of family, this study showed that the participants came from single family had more knowledge about TB disease, its agent, and treatment systems than who came from joint family. This may linked to parent’s income, education, media and other facilities. In single family, parents had more time to care their children and discuss about the general issues such as communicable diseases.

Almost all Bangladeshi has adopted with expanded program on immunization (EPI) and our study showed that with the success of EPI in Bangladesh, most of the respondents believed that BCG vaccination is effective against childhood TB. Of them, female were more aware than male. Previously, people in Bangladesh thought that TB is not curable. Now-a-days almost all Bangladeshi strongly believe that TB is a curable disease through ACSM activities of NTP. Most of the participants knew about the DOTs system for TB treatment, and the respondents who followed Hindu religion had more knowledge than Muslim. As public health concern, most of the respondents believed that TB is a major public health problem as it spread from person to person through coughing or sneezing.

## Conclusions

In this study 839 students were surveyed their knowledge about TB and established the association between the knowledge about TB and some selected socio-economic and demographic factors. This study revealed that more than half of students believed that TB is communicable disease, most of the students correctly answered about TB vaccination. More than two-third students agreed that TB is major health problem in Bangladesh. Most of the students believed that TB is curable disease, and about half of the students knew that bacteria are the agent for TB. A few students had idea about latent TB and most of the respondents had heard about DOTs program. Poor knowledge about TB was noted among the students who came from urban areas, and joint family. Students who were smoker and whose parents were higher educated had more knowledge on latent TB and curable respectively than their counterparts.

## Abbreviations

TB: Tuberculosis
WHO: World Health Organization
HIV: Human Immune Deficiency Virus
HBCs: High Burden Countries
DGHS: Directorate General of Health Services
NTP: National Tuberculosis Control Program
DR: Drug Resistant
DOT: Directly Observed Treatment
SPSS: Statistical Package for Social Sciences
TV: Television
BTV: Bangladesh Television
EPI: Expanded program on immunization
ACSM: Advocacy, Communication and Social Mobilization
